# Do Blood Group Antigens and the Red Cell Membrane Influence Human Immunodeficiency Virus Infection?

**DOI:** 10.3390/cells9040845

**Published:** 2020-03-31

**Authors:** Glenda M. Davison, Heather L. Hendrickse, Tandi E. Matsha

**Affiliations:** 1 SAMRC/CPUT/Cardiometabolic Health Research Unit, Department of Biomedical Sciences, Faculty of Health and Wellness Sciences, Cape Peninsula University of Technology, P.O. Box 1906, Bellville 7530, South Africa; hendricksehl@cput.ac.za (H.L.H.); matshat@cput.ac.za (T.E.M.); 2 Division of Haematology, Faculty of Health Science, University of Cape Town, Cape Town 7925, South Africa

**Keywords:** red cells, blood group antigens, Human Immunodeficiency Virus

## Abstract

The expression of blood group antigens varies across human populations and geographical regions due to natural selection and the influence of environment factors and disease. The red cell membrane is host to numerous surface antigens which are able to influence susceptibility to disease, by acting as receptors for pathogens, or by influencing the immune response. Investigations have shown that Human Immunodeficiency Virus (HIV) can bind and gain entry into erythrocytes, and therefore it is hypothesized that blood groups could play a role in this process. The ABO blood group has been well studied. However, its role in HIV susceptibility remains controversial, while other blood group antigens, and the secretor status of individuals, have been implicated. The Duffy antigen is a chemokine receptor that is important in the inflammatory response. Those who lack this antigen, and type as Duffy null, could therefore be susceptible to HIV infection, especially if associated with neutropenia. Other antigens including those in the Rh, Lutheran and OK blood group systems have all been shown to interact with HIV. More recently, experiments show that cells which overexpress the P^k^ antigen appear to be protected against infection. These reports all demonstrate that red cell antigens interact and influence HIV infection. However, as the red cell membrane is complex and the pathogenesis of HIV multi-factorial, the role of blood group antigens cannot be studied in isolation.

## 1. Introduction

It has been estimated that 37.9 million people carry Human Immunodeficiency Virus (HIV) and that Sub-Saharan Africa has the highest burden of infection [1]. Consequently, research on HIV has been extensive, and it has been well documented that the virus binds to the CD4 antigen and chemokine receptors (CXR4 and CCR5) in order to gain entry into cells and replicate [2]. Evidence has, however, emerged that the virus is also able to attach to CD4-negative cells such as erythrocytes. This was initially demonstrated in the presence of complement, and it was proposed, at that time, that the complement receptor 1(CR1) is the most likely mechanism of entry [3]. Others have gone on to reveal that in the absence of antibodies and complement, cell-free HIV was still capable of binding to red cells and, consequently, it was concluded that red cells, like dendritic cells, could act as a viral reservoir prior to the invasion of CD4+ cells [4]. Recently, further research has established that circulating immature CD71+ erythrocytes in HIV+ individuals are host to both intracellular and surface HIV particles, which significantly increases the movement, infectivity and replication of the virus [5]. These reports have all raised questions around the exact mechanism of HIV attachment and whether blood group antigens influence susceptibility.

The red cell membrane is host to approximately four hundred different surface antigens which have been grouped into thirty-six blood group systems [6,7]. The majority of these have been cloned and sequenced and have diverse functions, including structural, cellular transport and immune activation (Table 1) [6,7]. The distribution of blood groups varies between populations and geographical regions, and it has generally been accepted that this is due to natural selection, environmental factors and disease [8,9,10]. Blood group antigens are not only confined to red cells but can be found on other cells and tissue types, including epithelium and body fluids. This is dependent on the secretor status of individuals and the expression of the Fucosyltransferase 2 (FUT2) enzyme. Due to the diverse cellular and tissue expression of ABO antigens, they have been associated with many conditions including cardiovascular disease, venous thrombosis and malignancy [10,11,12,13].

The presence or absence of red cell antigens has also been implicated in the susceptibility and pathogenesis of bacterial, viral and parasitic infections. These include *Vibrio cholera* [14], *Escherichia coli* [15], *Helicobacter pylori* [16], Norovirus [17], Rotavirus [18], and Human Immunodeficiency Virus (HIV) [6,19]. A well-described example is the Duffy antigen, which has been identified as a receptor for the parasite *Plasmodium vivax.* In Sub-Saharan Africa, where the majority of the population phenotype as Duffy null (Fy(a-b-)) and do not express this antigen, many individuals are protected against both *Plasmodium vivax* and *Plasmodium knowlesi* [20]. 

These reports all provide evidence that blood group antigens could influence the pathogenesis of infectious disease including HIV. Despite this, the relationship between these red cell surface antigens and HIV still remains complex, controversial and not fully explained. 

With this in mind, the main objective of this review was to re-examine the current knowledge on this subject in an attempt to provide further clarity on the association between HIV and red cell antigens.

## 2. Human Immunodeficiency Virus

Human Immunodeficiency Virus (HIV) belongs to the family of *Retroviridae* and the genus *Lentivirus*. The structure of both the HIV-1 and HIV-2 strains is similar, and both possess a lipid-protein membrane. Embedded in the membrane is glycoprotein gp41, which is attached to gp120. Both surface proteins are encoded by the *env* gene and are vital in attaching the virus to the host cell by binding to cell surface receptors. Beneath the surface membrane is p17, a matrix protein that is encoded by the *gag* gene. This gene also encodes the core proteins p24 and p6 and the nucleocapsid protein p7 which is bound to viral RNA [21,22].

The virus contains two strands of RNA, together with the enzymes, protease, integrase, and reverse transcriptase—all of which are essential in the replication process, and are encoded by the *pol* gene. Other proteins such as Tat, promote transcription, while Rev is responsible for the transport of messenger RNA from the nucleus to the cytoplasm. Vpr, another protein, ensures that the reverse transcribed DNA is able to access the nucleus of non-dividing cells, while signal transduction and the down-regulation of the CD4 antigen, in order to allow the budding process to take place, is facilitated by the viral protein known as Nef [21,22].

### HIV Replication

Replication is initiated upon the attachment of the virus to the host cell. The proteins gp120 and gp41 are essential for this to occur and it begins with gp120 binding to the CD4 molecule, which is expressed on T helper cells, macrophages, eosinophils and dendritic cells [23]. Once this has taken place, a structural change occurs which allows gp120 to bind the chemokine receptors CXR4 and CCR5, on the cell surface. The natural ligands of CCR5, RANTES and MIP-1-α and β, are potentially able to suppress HIV infection and have been shown to compete with HIV for binding [24].

After attachment, gp41 penetrates the host cell membrane and forms a loop structure that allows the virus to enter the cell. The viral RNA is released into the cytoplasm and is converted by reverse transcription into pro-viral DNA. After double-stranded DNA is formed, it is integrated into the genome of the host cell by the enzyme Integrase [25].

Recent investigations, utilizing multi-color single-molecule localization microscopy (SMLM) and two-color photoactivated localization microscopy (PALM), have demonstrated that gp41 is also able to interact with the T-cell receptor (TCR) on CD4+ T-cells. This interaction was shown to lead to early activation of the cell, with phosphorylation of the TCR, upregulation of CD69 and cell death after approximately 24 h [26]. This information is important as once the activation of the target cell occurs, the pro-viral DNA is transcribed into messenger RNA and the synthesis of new viral proteins begins. The new viruses move toward the cell surface and are released into the circulation by a process called “budding”. During this process, HIV incorporates many of the host cell proteins, including ABO carbohydrate molecules, which become part of the surface membrane [27].

## 3. The ABO Blood Group System

The ABO blood group system has evolved over millions of years and at least two theories on how this occurred have been proposed. One hypothesis is that group O was the original blood group and that groups A and B were the results of mutations that occurred due to population migration and environmental factors. This has been supported by the high prevalence of group O amongst the earliest human populations [12,28,29,30]. In contrast, others suggest that group O, which results from the inactivation of the A1 glycosyltransferase gene, evolved from a common group AB due to the selective advantage and natural resistance of Group O individuals to the malaria parasite. This has been supported by observations that individuals with blood type O are protected against severe malaria due to decreased red cell rosetting and adherence [20,31,32]. This evolutionary history is relevant as it illustrates the relationship of blood groups with the environment, and how the expression of blood group antigens has evolved in response to diseases.

The *ABO* gene does not code directly for ABO antigens but for specific enzymes called glycosyltransferases, which transfer *N*-galactosamine (group A) or D-galactose (group B) to the H antigen to form the A and B blood group antigens. This does not occur in group O individuals and their red cells express the H antigen [9]. The resulting A, B and H antigens contain a lipophilic section which is attached to the red cell membrane and a carbohydrate chain which protrudes above the cell surface [6]. The exact function of the ABO system remains unclear. However, in red cells, they appear to play a role in the formation of the glycocalyx [8]. During early life, after exposure to the environment, the immune system forms naturally occurring antibodies against those A and B antigens which are missing, while both anti-A and anti-B antibodies are present in Group O. These are powerful IgM antibodies that cause blood transfusion reactions if incompatible blood is transfused [6,9,31,32].

The influence of the ABO blood group system on the pathogenesis of disease has been described by many researchers and its role in the susceptibility or protection against HIV has been widely debated [6]. It has been suggested that polymorphic blood group antigens expressed on the surfaces of epithelia, red blood cells, platelets, and granulocytes are utilized as attachment receptors by HIV and that anti-A and/or anti-B naturally occurring antibodies could confer protection by neutralizing the virus [33]. Evidence for these hypotheses has been provided by studies which have shown that mononuclear cells and lymphocytes can adsorb ABO antigens from the surrounding plasma and that, during the “budding process”, HIV can incorporate these antigens onto the viral surface, as they have been found bound to gp120 [27]. Further in vitro investigations have gone on to demonstrate that the viral bound ABO antigens result in HIV becoming sensitized to naturally occurring, complement-fixing IgM antibodies which could neutralize the virus [27,34]. These experiments have led to the hypothesis that naturally occurring ABO antibodies could be part of the innate immune response and may provide protection when the virus is transmitted between individuals who have different blood groups [35]. Despite these reports, the role of the ABO system in the pathogenesis and susceptibility to HIV infection has remained unclear, initiating further population-based studies.

However, investigations examining the relationship between ABO antigens and HIV infection still remain controversial. Early studies in India reported a high incidence of HIV positivity among Group O individuals, but these results could not produce statistically reliable conclusions [36] and have been challenged by others who have demonstrated significantly decreased HIV positivity among Group O individuals [37]. Others have reported higher HIV-2 infection rates amongst those who are AB positive [38] and in Brazil, a higher frequency of HIV was observed amongst those who were group B [39]. Furthermore, a number of studies could not demonstrate any association between ABO antigen expression and HIV infection [19,40,41] and concluded that this blood group system probably does not influence susceptibility. The majority of these reports were superficial and did not include large numbers of participants or account for population and ethnic differences. Therefore, clarity on the ABO blood group and HIV infection still remains vague and debatable.

Other researchers, realizing that the influence of the ABO blood group is multi-factorial and probably involves other factors, went on to examine its association with the CD4 count during the course of the disease. This investigation demonstrated that HIV patients with blood groups A and AB maintained significantly higher CD4 counts than the other blood groups [42]. These authors again suggested a role for the ABO antigens in the immune response and proposed that ABO antigens may be more important in the progression of the disease rather than in initial infection. Although these findings added interesting information, they did not conclusively link HIV to the ABO blood group system, and so it is clear that further research with larger numbers and more rigorous analysis is needed to fully answer the question.

ABO blood group antigens are also found on the surface of mucosal epithelial tissue and in body fluids including the genital tract. This is dependent on the activity of the enzyme, Fucosyltransferase 2 (FUT2), which is responsible for ABO biosynthesis in body fluids [43]. Approximately 20–30% of people do not synthesize this enzyme, are unable to express blood group antigens in body fluids and are termed non-secretors. The secretor status of individuals has been shown to influence other viral infections, such as the *norovirus,* which binds to antigens expressed on mucosal surfaces of the gastrointestinal tract [44,45,46]. This association of FUT2 and disease has led to the hypothesis that non-secretors could be protected against HIV infection [47].

An early study conducted on 219 heterosexual individuals revealed a significantly higher incidence of secretors among those who had acquired HIV through sexual intercourse compared to those who had acquired it through intravenous drug use. This implied that the expression of ABO antigens on mucosal surfaces could influence infection [48]. This early work has been supported by three independent studies conducted in Senegal, Kenya, and Nigeria. In each of these, it was demonstrated that individuals who were secretors had a higher incidence of HIV positivity and that those who did not have the FUT2 enzyme appeared to have a certain amount of protection. It was hypothesized that this phenomenon could be due to the slower progression of the disease [45,47]. Although these studies are of interest, once again, they are not conclusive and therefore confirmation by larger more comprehensive research is still required.

Despite a number of investigators attempting to study and verify the link between ABO blood group antigens and HIV infection, the number of authors claiming an association is similar to those who have concluded that there is no relationship. It is therefore clear that more in depth rigorous research, across larger populations and not just small localized groups, is required before this question can be fully answered.

## 4. The Duffy Blood Group System

The Duffy blood group antigen, also known as the Duffy antigen receptor for chemokines (DARC), is a large multi-pass protein that spans the red cell membrane seven times and has a 62 amino acid extracellular N-terminal domain [6]. Its expression is controlled by two alleles (FY*1 and FY*2) which encode the FY^a^ and FY^b^ antigens [7,31] and it can be found on the surface of red cells and other tissue such as brain, kidney and endothelium [49].

DARC functions as a chemokine receptor, binds a wide range of inflammatory cytokines including interleukin 8, RANTES and monocyte chemoattractant protein (MCP-1) and is thought to modulate the immune response [50]. The majority of individuals descended from Africa do not express the DARC antigen on their red cells and have the Duffy null phenotype ((Fy(a-b-)) which provides protection to infection by the *Plasmodium vivax* and *Plasmodium knowlesi* malaria parasite. The lack of DARC expression in these individuals is due to a polymorphism in the *FY* gene promoter region which affects the binding site for the GATA transcription factor in erythrocytes, but not in other cells such as those of the endothelium [51]. As a chemokine receptor, DARC is able to bind HIV-2 but not HIV-1 and may, therefore, influence both initial infection, replication and progression [50,52].

Like the ABO blood group, the relationship between the Duffy antigen and HIV remains controversial. It has, however, been established that Duffy-null individuals have lower levels of inflammatory chemokines such as MCP-1 in their plasma [50]. These cytokines play an important role in the immune response and are able to inhibit HIV infection, and therefore a disruption in their balance could influence susceptibility to the virus. This finding, together with the fact that certain strains of the virus are able to attach to DARC, seemed to suggest that those who are Duffy null could be more susceptible to HIV infection.

A study of African Americans, in which the relationship between DARC expression and HIV was investigated, seemed to support this proposal. The results revealed that those with the Duffy null phenotype were more likely to be infected; however, more importantly, these individuals appeared to have a significantly slower progression [53]. Similar results were reported in 2009 [54] and, although many questions remained unanswered, it appeared that the lack of the Duffy antigen influenced the interaction of the virus with chemokines and that this consequently interfered with the balance and process of inflammation [51].

Benign Ethnic Neutropenia (BEN) is associated with the Duffy null phenotype and, as neutrophils are part of the innate immune system, it has been hypothesized that there could be a relationship between BEN, the Duffy antigen and HIV. A study of 142 high-risk sex workers in South Africa appeared to confirm this, as those who had both the Duffy null phenotype and associated neutropenia had a higher seroconversion rate, which implied that the Duffy null phenotype alone did not increase susceptibility [55]. This report, although providing interesting new knowledge, has not been verified and again, further in depth investigations which explore the mechanisms of this phenomenon are required.

Despite the evidence supporting the relationship between the Duffy null phenotype and HIV, there has been an equal number of studies that have contradicted these findings and have reported no significant association [19,37,56]. Two of these were conducted in Sub-Saharan Africa on small numbers of patients using phenotypic assays [19,37], while the third examined the genotype of 454 HIV+ and 425 HIV- African Americans [56]. In all three investigations, the difference between the HIV+ and HIV- groups were insignificant. 

These reports all imply that the relationship between the Duffy blood group system and HIV is far more complex than it appears and that more comprehensive investigation and research is required. However, as the Duffy blood antigen plays a significant role in the inflammatory process and functions as a chemokine receptor, the hypothesis that this antigen influences the evolution of HIV infection remains reasonable and therefore more comprehensive investigation is recommended.

## 5. The Rh Blood Group

The Rh blood group system antigens are highly immunogenic and are encoded by the *RHD* and *RHCE* genes. A total of 49 antigens have been identified—of which, D (RH1), C (RH2), E (RH3), c (RH4), and e (RH5) are the most common. The Rh antigen is a complex multi-pass protein which transverses the cell membrane 12 times. A proportion of the human population lacks the D antigen, which is a result of a deletion of the *RHD* gene. The function of the Rh antigens remains vague. However, research has shown that they are associated with the maintenance of red cell integrity and the band 3 protein structure and that they play a role in ammonium transport [8].

Studies examining the Rh blood group system in HIV are scarce and contradictory [36,38]. The most probable reason for this is the low numbers of HIV-positive participants in comparison to controls which has made statistically significant conclusions difficult. However, a study carried out on 346 HIV-negative and 196 HIV-positive Botswanans found that the expression of Rh antigen C (RH2) and E (RH3) was significantly decreased in those who were HIV positive, implying that they could have a protective role. However, in order to further interrogate this result, binary logistic regression analysis was used to analyze the interaction of all significant blood groups. Following this, only the C antigen maintained its significance, implying that it could have a protective effect [19].

A further investigation on a similar number of participants from South Africa (119 HIV positive vs. 317 HIV negative) examined both Rh antigen expression and genotype. This study did not confirm the findings from Botswana but did report that none of the HIV-positive individuals were RH1 negative or had the genotype *rr.* [37]. This study however, had a significant number of limitations, including the lack of demographic data and the very low number of individuals who had the *rr* genotype. This made any conclusions on the influence of the Rh antigen unreliable.

Although these two reports from Southern Africa seemed to imply that the Rh blood group system may play a role in the pathogenesis of the virus, both studies were superficial and therefore inconclusive. The relationship of the Rh blood group system with HIV and the mechanism of interaction, if any, still remains purely speculation.

## 6. The P^k^ Antigen

When first described, the P blood group consisted of three antigens (P, P1 and P^k^) and, although the P1 antigen is exclusive to red cells, P^k^ and P can be expressed on other cells such as lymphocytes, platelets, and endothelium. Over time, the P blood group has been expanded and is now recognized as three separate blood group systems (PIPK, Globoside and Forssman) with six different antigens [6]. The presence of these antigens in the urinary tract has been strongly associated with *E. coli* infections [6] and therefore this blood group may be a candidate for investigation in the context of HIV. A study in Botswana demonstrated that red cell-bound P1 antigen was associated with a significantly higher risk of HIV infection [19] and other studies have focused on the P^k^ antigen [57].

Glycosphingolipids are able to bind to HIVgp120 and are therefore associated with the binding of viruses to host cells [6,57]. The P^k^ antigen is a glycosphingolipid and it has been shown that in Fabrys disease, which has increased amounts of the P^k^ antigen within cells, HIV-1 is inhibited [57]. This has led to investigations that have focused specifically on the P^k^ antigen.

Experiments were conducted in which the resistance or susceptibility to HIV infection was compared between peripheral blood mononuclear cells which overexpressed P^k^ to those which had no expression. Those which lacked P^k^ on their surface had significantly increased susceptibility to HIV, and this finding was confirmed in genetically manipulated CD4+ HeLa cells [10]. These in vitro experiments imply that P^k^ could be important in the protection against HIV infection and it is thought that the antigen competes with CCR5 and CXCR4 in binding to HIVgp120. When P^k^ is highly expressed, HIV will preferentially attach to it, preventing fusion and penetration of the cell [10]. The P^k^ antigen is a very rare blood group antigen, making large studies difficult. However, these findings could assist in developing therapies for the prevention of infection.

## 7. Other Blood Group Antigens

The Lutheran blood group antigens transverse the red cell membrane once, are members of the immunoglobulin superfamily and function as laminin receptors [58]. In patients with sickle cell disease, the red cell membrane has increased surface expression of Lutheran antigens and it is hypothesized that they could play a role in the adherence of the sickle cells to the endothelial cell wall [8]. The expression of the Lutheran antigens is regulated by the *in(Lu)* gene which also influences the expression of other associated antigens such as CD44. CD44 is an adhesion molecule and plays a role in the homing of lymphocytes and can influence the immune response [58]. It is therefore very possible that this complex blood group system could play a role in the pathogenesis of HIV. Not much has been published about this blood group. However, a study performed in Botswana showed that the expression of the Lu^b^ antigen was significantly more prevalent in patients with HIV, despite the prevalence of this blood group not being any different between the general Botswana population and other African nations [19]. It must, however, be pointed out that this is a single isolated report and before any conclusions can be made, further work is recommended.

CD147, alternatively known as Basigin, belongs to the OK blood group system and is also a member of the immunoglobulin superfamily. It is important in red cell trafficking, cell adhesion, and inflammation, and has been associated with *Plasmodium falciparum* and HIV [6]. CD147 is a receptor for cyclophilin A, which if integrated into HIV, significantly increases its infectivity. In a study using a cell line transfected with CD147, it was shown that the transfected cells were significantly more sensitive to the virus than controls. After treating the cells with an anti-CD147 antibody, they became resistant to infection. These studies led to the conclusion that cyclophilin A bound to the CD147 molecule could explain the increased sensitivity [59].

Both these blood group systems influence the immune response and inflammation, and therefore it is plausible that they could have an impact on HIV infection. However, the evidence supporting this remains very scarce and therefore inconclusive.

## 8. Conclusions

The main objective of this short review was to provide an update on the association of blood group antigens and the pathogenesis of HIV infection. Although much work has been completed in this area, it is clear that blood groups cannot be studied in isolation, as the function and interaction of red cell surface antigens with other cells and molecules, including pathogens, is multi-faceted.

The red cell membrane is highly complex, and the proteins on its surface interact with many other molecules and extracellular substances. Furthermore, the pathogenesis of infectious disease, and in particular HIV, is intricate and involves not only the binding and fusion of the virus with target cells but also interaction with both the innate and adaptive immune response. These complexities, together with the distribution and genetic diversity of blood group antigen expression, make research in this area challenging.

This mini-review has demonstrated that there is some evidence which suggests that blood group antigens and red blood cells play a role in the susceptibility or protection against HIV infection. However, much of the previous work is superficial and has thus generated more questions than answers. The association of blood group antigens and HIV therefore remains a much neglected area of biological research and further large population studies using high-throughput sequencing to analyze all polymorphic blood groups are required to add further knowledge to this subject.

## Figures and Tables

**Table 1:** Blood group systems recognized by the International Society of Blood Transfusion.

System	Chromosome	Gene	Type	Function
ABO	9q34.2	*ABO*	Carbohydrate	Glycosyltransferase
MNS	4q31.21	*GYPA* *GYPB*	Single-pass protein	Single-pass protein, band 3 ankyrin complex, red cell zeta potential
PIPK	22q13.2	*A4GALT1*	Carbohydrate	α1,4 Galactosyltransferase
Rh	1p36.11	*RHD* *RHCE*	Multi-pass protein	Ammonium transport, band 3 metabolism
Lutheran	19q13.32	*LU*	Single-pass protein	Laminin receptor
Kell	7q34	*KEL*	Single-pass protein	Endothelin 3-converting enzyme
Lewis	19p13.3	*FUT3*	Carbohydrate	α¾-Fucosyltransferase
Duffy	1q23.2	*DARC*	Multi-pass protein	Chemokine receptor
Kidd	18q12.3	*SLC14A1*	Multi-pass protein	Urea transport
Diego	17q21.31	*SLC4A1*	Multi-pass protein	Band 3/anion exchange/cytoskeleton stability
Cartwright/Yt	7q22.1	*ACHE*	GPI-linked protein	Acetylcholinesterase
Xg	Xp22.33	*XG, MIC2*	Single-pass protein	Adhesion molecule
Scianna	1p34.2	*ERMAP*	Single-pass protein	Unknown
Dombrock	12p12.3	*ART4*	GPI-linked protein	ADP-ribosyltransferase
Colton	7p14.3	*AQP1*	Multi-pass protein	Water transport/band 3 metabolism
Landsteiner–Wiener	19p13.2	*ICAM4*	Single-pass protein	Adhesion molecule
Chido/Rodgers	6p21.3	*C4A, C4B*	Absorbed from plasma	Complement, C4
H (SE)	19q13.33	*FUT1* *FUT2*	Carbohydrate	α1,2-Fucosyltransferase, type 2 H antigen α1,2-Fucosyltransferase, type 1, 3 and 4, secretor (ABH)
Kx	Xp21.1	*XK*	Multi-pass protein	Unknown
Gerbich	2q14.3	*GYPC*	Single-pass protein	Glycophorins C and D, membrane stability
Cromer	1q32.2	*CD55*	GPI-linked protein	Complement regulation
Knops	1q32.2	*CR1*	Single-pass protein	Complement regulation
Indian	11p13	*CD44*	Single-pass protein	Cell adhesion
Ok	19p13.3	*BSG*	Single-pass protein	Cell trafficking, cytophillin receptor, adhesion and signaling
Raph	11p15.5	*CD151*	Multi-pass protein	Cell adhesion
John Milton Hagan	15q21.1	*SEMA7A*	GPI-linked protein	T-cell-mediated inflammation, integrin receptor
I	6p24.2	*GCNT2*	Carbohydrate	β1,6-N-acetylglucosaminyltransferase
Globoside	3q26.1	*B3GALNT1*	Carbohydrate	β1,6-N-acetylgalactosaminyltransferase
Gill	9p13.3	*AQP3*	Multi-pass protein	Water, glycerol, peroxide transport
Rh-Associated glycoprotein	6p21	*RHAG*	Multi-pass protein	Ammonium transport—associated RhD and RhCE
FORS	9q34.13	*GBGT1*	Carbohydrate	α1,3-N-acetylgalactosaminyltransferase
JR	4p22	*ABCG2*	Multi-pass protein	ATP-dependent transport
Lan	2q36	*ABCG6*	Multi-pass protein	Porphyrin and heme transport
Vel CD59 Augustine KANNO Sid	1p36.32 11p13 6p21.1 20p13 17q21.32	*SMIM1* *CD59* *SLC29A1* *PRNP* *B4GALNT2*	Single-pass protein GPI-linked protein Multi-pass protein Prion protein	Red cell formation regulation Complement regulation Protein transporter Acetylgalactosaminyltransferase
